# L-Carnitine Supports the In Vitro Growth of Buffalo Oocytes

**DOI:** 10.3390/ani12151957

**Published:** 2022-08-02

**Authors:** Avijit Kumar Modak, Md Hasanur Alam, Md Nuronnabi Islam, Nipa Paul, Ireen Akter, Md Abul Hashem, AKM Ahsan Kabir, Mohammad Moniruzzaman

**Affiliations:** Department of Animal Science, Bangladesh Agricultural University, Mymensingh 2202, Bangladesh; avimodak@yahoo.com (A.K.M.); hasanur@bau.edu.bd (M.H.A.); sagor.as@bau.edu.bd (M.N.I.); nipapaul092@gmail.com (N.P.); akterireen8@gmail.com (I.A.); hashem_as@bau.edu.bd (M.A.H.); ahsankabiras@bau.edu.bd (A.K.M.A.K.)

**Keywords:** buffalo, in vitro growth, L-carnitine, maturation, oocytes

## Abstract

**Simple Summary:**

The objective of this study was to examine the effect of L-carnitine on the growth and subsequent nuclear maturation of buffalo growing oocytes in vitro. Oocyte-granulosa cell complexes (OGCs) were collected from early antral follicles of slaughtered buffaloes and cultured in in vitro growth (IVG) medium with the supplementation of different concentrations (0, 1.25, 1.875 or 2.5 mM) of L-carnitine for 6 days. The results showed that L-carnitine increased the diameter of buffalo oocytes in vitro. L-carnitine also enhanced the antrum-like structure formation and prevented the degeneration of buffalo oocytes in culture. Furthermore, L-carnitine-treated oocytes showed a higher rate of nuclear maturation up to the metaphase II (MII) stage and a lower rate of degeneration. In conclusion, L-carnitine enhances the growth, prevents degeneration, promotes the formation of antrum-like structures and supports nuclear maturation of buffalo oocytes in vitro.

**Abstract:**

This study aimed to determine the effect of L-carnitine on the growth and subsequent nuclear maturation of buffalo small growing oocytes (92–108 µm in diameter) in vitro. Oocyte-granulosa cell complexes (OGCs) were dissected from early antral follicles of slaughtered buffaloes and cultured in in vitro growth (IVG) medium with the supplementation of different concentrations (0, 1.25, 1.875 or 2.5 mM) of L-carnitine for 6 days. The results revealed that L-carnitine increased the diameter of buffalo oocytes in vitro. The degeneration rate was significantly (*p* < 0.05) lower in 2.5 mM of L-carnitine-treated oocytes (10%) than others (55%, 45% and 32.5% in 0, 1.25 and 1.875 mM of L-carnitine-supplemented groups, respectively). The OGCs showed antrum-like structures significantly (*p* < 0.05) higher in the 2.5 mM of L-carnitine group (74.0%) than the 0- and 1.25-mM groups (34.6% and 38.1%, respectively). Furthermore, in vitro grown oocytes were placed in in vitro maturation (IVM) medium for 24 h to examine meiotic competence of in vitro grown oocytes with L-carnitine. The L-carnitine (1.875 and 2.5 mM) treated oocytes showed a higher rate of nuclear maturation up to the metaphase II (MII) stage and a lower rate of degeneration. In conclusion, L-carnitine enhances the growth, prevents degeneration, promotes the formation of antrum-like structures and supports nuclear maturation of buffalo oocytes in vitro.

## 1. Introduction

The buffalo is an important livestock resource in many countries, particularly in Asia and the Mediterranean regions of Europe. They are renowned for their high feed conversion efficiency and high disease resistance [1,2]. Their high butter fat content in milk, low maintenance requirement and high draught output make buffaloes popular [1]. Despite these qualities, buffaloes have poor reproductive performance; poor expression of estrous signs, long post-partum ovarian inactivity, late pubertal maturity, poor conception rate and long calving intervals [2,3,4]. Various assisted reproductive technologies (such as artificial insemination, estrous synchronization, super ovulation, etc.) are still challenging in buffaloes [5,6]. 

Mammalian ovaries contain a huge number of small oocytes at the growing stage. Many approaches have been made to grow these small oocytes in vitro. A lot of growth culture systems have been practiced making use of bovine small growing oocytes (<100 µm in diameter) [7,8,9,10]. A long-term in vitro growth culture system for bovine small growing oocytes has been developed by Hirao et al. [9]. In their growth culture system, oocyte-granulosa cell complexes were cultured in medium supplemented with 4% (*w*/*v*) polyvinylpyrrolidone for two weeks. This system supported the growth and development of bovine small growing oocytes (<100 µm in diameter), and the oocytes-granulosa cell complexes (OGCs) developed an antrum structure during culture. Follicular antrum is the special structure in mammalian ovaries. Antrum formation seems to ensure the mammalian viviparity and long reproductive lifespan, and to be crucial for female reproduction in mammals. However, some of the growth culture systems used for bovine small growing oocytes have resulted in the successful production of offspring [7,8,9,10]. Oocytes displayed a size-dependent ability to undergo meiotic maturation and transcriptional activity [11,12]. Oocytes with a diameter of <115 µm resumed meiosis during in vitro maturation but they are not competent to mature to metaphase II both in buffalo [12] and bovine [13]. To confer full maturational competence onto these oocytes, culture systems which can support their growth are needed.

L-carnitine is a naturally produced, vitamin-like, water-soluble quaternary ammonium compound. The biologically active form of carnitine (3-hydroxy-4-N-trimethyl amino butyrate, C_7_H_15_NO_3_) is known as L-carnitine. It is mainly synthesized from lysine and methionine in the liver. L-carnitine is required for the transportation of fatty acids from the cytosol to the mitochondria to generate metabolic energy during the breakdown of lipids (fats). As an antioxidant, L-carnitine neutralizes free radicals, especially superoxide anions, and protects cells from oxidative damage-induced apoptosis [14]. L-carnitine have a key role in ATP production through β-oxidation by transferring the fatty acids into mitochondria during in vitro oocyte maturation, thus promoting oocyte maturation and embryonic development in bovine [15], porcine [16] and buffalo [17]. The antioxidant properties of L-carnitine reduce cell apoptosis [18,19]. In vitro growth of porcine growing oocytes with L-carnitine decreased apoptosis of granulosa cells improves mitochondrial activity and meiotic competence [20]. However, so far, the effects of L-carnitine on the in vitro growth of buffalo small growing oocytes are not yet clear. Hence, this study aimed to examine the effects of L-carnitine in in vitro growth culture medium on antrum formation, oocyte growth and the maturation rate of buffalo small growing oocytes. 

## 2. Materials and Methods

Slaughterhouse-derived buffalo oocytes were cultured with the supplementation of L-carnitine to examine its effects on antrum formation, oocyte growth, and maturation rate. Unless otherwise stated, all chemicals were purchased from Sigma-Aldrich (St. Louis, MO, USA).

### 2.1. Collection of Oocytes-Granulosa Cell Complexes (OGCs)

Buffalo ovaries were collected from a slaughterhouse and transported to the laboratory in a thermo flask in physiological saline (0.9% [*w*/*v*] NaCl) at room temperature (25–30 °C). The ovaries were collected during autumn and winter seasons (September-December). The collected ovaries were washed at least five times in physiological saline and trimmed to remove the surrounding adipose tissues and overlying bursa. Ovaries were washed with physiological saline for a further five times. Early antral follicles were collected mechanically according to the procedure described previously [21]. In brief, ovarian cortex were sliced into small pieces (around 2 mm in thickness) using a surgical blade and a pair of forceps. Early antral follicles (1.0–1.5 mm in diameter) were isolated from the slices under a zoom stereo microscope (CZM6, Labomed, CA, USA) using a pair of forceps. The follicles were kept in a small Petri dish containing TCM-199 supplemented with 0.32% (*w*/*v*) bovine serum albumin (BSA) and 250 µg/mL gentamycin sulfate at room temperature (25–30 °C). The follicles were gently opened under a zoom stereo microscope for the collection of oocyte-granulosa cell complexes (OGCs). OGCs were kept in a culture dish (No. 1008, Falcon, Becton Dickinson and Company, Franklin Lakes, NJ, USA) containing 2 mL of TCM-199 supplemented with 0.32% BSA and 250 µg/mL gentamycin sulfate until further use. On the other hand, cumulus-oocyte complexes (COCs) were collected by aspiration of 4–8 mm follicles using a syringe (Henke Sass Wolf, Tuttlingen, Germany) with an 18 g needle containing 1–2 mL of aspiration medium (TCM-199 supplemented with 3.2 mg/mL bovine serum albumin and 250 µg/mL of gentamycin sulfate), considered as in vivo grown oocytes.

### 2.2. In Vitro Growth (IVG) of Oocytes

In vitro growth of oocytes was performed as described by Hirao et al. [9]. OGCs were cultured for 6 days in 200 μL of culture medium in a 96-well culture dish (Biocoat Collagen I Cell ware; Becton Dickinson Biosciences, San Jose, CA, USA) under an atmosphere of 5% CO_2_ in humidified air at 38.5 °C. OGCs were placed individually into each well of a 96-well culture dish for incubation. The basic IVG medium was TCM-199 supplemented with 5% (*v*/*v*) fetal bovine serum (FBS; Gibco BRL, Grand Island, NY, USA), 50 µg/mL L-ascorbic acid, 4% (*w*/*v*) polyvinylpyrrolidone (molecular weight 360,000), 1 mM sodium pyruvate (29806-54; Nacalai Tesque, Inc., Kyoto, Japan), 4 mM hypoxanthine, 55 µg/mL cysteamine, 0.05 µM dexamethasone, 10 ng/mL 17 β–estradiol and 10 ng/mL androstenedione, as described in our previous report [21]. To evaluate the effects of L-carnitine the basic IVG medium was supplemented with different concentrations (0, 1.25, 1.875 or 2.50 mM) of L-carnitine (C0283). TCM-199 were used to dilute and make stock solution for L-carnitine. The day of OGCs collection was designated as day 0. The diameter of oocytes (excluding the zona pellucida) were measured using an ocular micrometer attached to a phase contrast inverted microscope (TCM 400; Labomed, CA, USA) at day 0, day 3 and day 6. The diameter of oocytes at Day 0 ranged between 92 and 108 μm, which are thought to still be in the growing phase without fully maturational competence [11,12]. Half of the medium (100 µL) was replaced with new medium at day 3 and day 5. Formations of antrum-like structures (formation of multiple spaces between granulosa cells) were observed under a microscope at day 3 and day 6 of culture. OGCs with cytoplasmic degeneration and detachment of cumulus cells were classified as degenerated oocytes. After 6 days of culture, only healthy OGCs were subjected to in vitro maturation.

### 2.3. In Vitro Maturation (IVM) of Oocytes

Both in vitro grown healthy oocytes and collected in vivo grown oocytes were washed at least three times with TCM-199 supplemented with 8% (*v*/*v*) fetal bovine serum (FBS; Gibco BRL, Grand Island, NY, USA), 0.8 mg/mL sodium pyruvate, 1 mM L-glutamine and 50 µg/mL gentamycin sulfate. The oocytes (4–5 oocytes in each droplet) were transferred to IVM droplets (100 μL) for in vitro maturation for 24 h under an atmosphere of 5% CO_2_ in humidified air at 38.5 °C. The IVM medium was TCM-199 supplemented with 10% (*v*/*v*) FBS, 5 μg/mL porcine follicle stimulating hormone (FSH; NIDDK, Washington, DC, USA), 0.1 mg/mL Na-pyruvate, 5 μg/mL 17 β-estradiol and 0.08 mg/mL gentamycin sulfate, according to the method described by Taketsuru et al. [22].

### 2.4. Assessment of Oocyte Maturation

After IVM, oocytes were washed in physiological saline and denudated mechanically using a Pasteur pipette with the help of 0.1% (*w*/*v*) hyaluronidase enzyme. The oocytes were fixed in aceto-ethanol (Acetic Acid: Ethanol = 1:3) for 48 h. The oocytes were stained with 1% (*w*/*v*) aceto-orcein for 5 min and washed with aceto-glycerol (Glycerol: Acetic Acid: Double Distilled Water = 1:1:3). Oocytes were then examined to assess the nuclear maturation under a differential interference contrast (DIC) microscopy (Olympus Corporation, Center Valley, PA, USA). The oocytes were categorized according to their nuclear maturation as described previously [13,23]. In this experiment, the oocytes after meiotic resumption were categorized as diakinesis (DK), metaphase I (MI) and metaphase II (MII). Oocytes with pyknosis, large vacuoles, condensed cytoplasm, disappearing nuclear membranes, shrinkage of nucleus and dissociation of the nuclear membrane were recognized as degenerating oocytes, according to our previous report [21].

### 2.5. Statistical Analysis

All data from at least five replications were represented as mean ± SEM (standard error of mean). The data were analyzed using one-way ANOVA followed by Tukey’s test (IBM SPSS Statistics, version 22, Endicott, NY, USA). Differences at *p* < 0.05 were considered statistically significant.

## 3. Results

### 3.1. L-Carnitine Enhances the Growth of Buffalo Oocytes In Vitro

**Figure 1:**
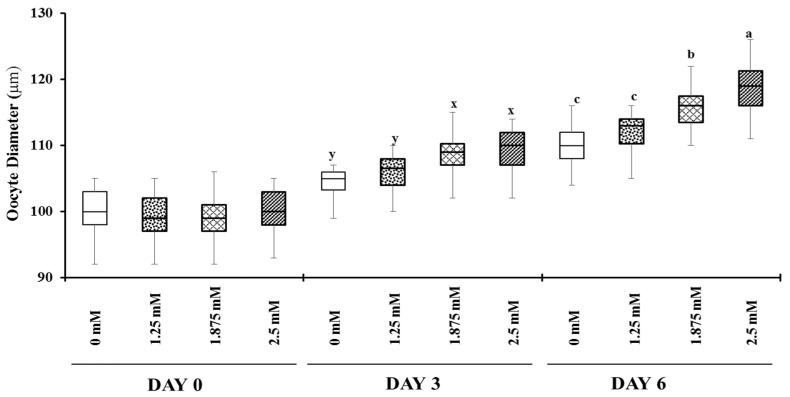
Effect of L-carnitine on oocyte diameters during in vitro growth culture for 6 days. The IVG media was supplemented with 0, 1.25, 1.875- and 2.5-mM L-carnitine and diameter of oocytes observed at day 0, day 3 and day 6. Values with different superscripts (a–c and x–y) in the same days differed (*p* < 0.05).

The changes in oocyte diameter over period of growth culture are shown in Figure 1. The diameters of oocytes at day 0 were 100.0 ± 0.5, 99.4 ± 0.5, 99.1 ± 0.5 and 100.1 ± 0.5 μm in 0, 1.25, 1.875 and 2.5 mM of L-carnitine-supplemented groups, respectively. Oocyte diameters significantly increased in 1.875 and 2.5 mM of L-carnitine than 0 and 1.25 mM of L-carnitine-treated groups on day 3 and day 6. On day 3 of culture, there were no significant differences (*p* < 0.05) in oocyte diameters between 1.875 and 2.5 mM of L-carnitine-treated groups. Oocyte diameters were higher in 2.5 mM of the L-carnitine (118.8 ± 0.5) group than the 1.875 mM (115.8 ± 0.4) group at day 6. These results show that L-carnitine supports the growth of buffalo oocytes in vitro at 1.875- and 2.5-mM levels.

### 3.2. L-Carnitine Reduces Oocyte Degeneration 

The numbers of degenerated oocytes were examined at day 0, 3 and 6 among the different treatment groups (Table 1). On day 3, 45%, 40%, 30% and 7.5% oocytes were degenerated in 0, 1.25, 1.875 and 2.5 mM of L-carnitine-supplemented groups, respectively. A significantly (*p* < 0.05) higher percentage of surviving oocytes were recorded in the 2.5 mM L-carnitine group than others on day 3 and 6. Moreover, the higher percentages of degeneration rate were recorded in 0 and 1.25 mM of L-carnitine groups (55% and 45%) than the 1.875 and 2.5 mM (32.5% and 10%) groups on day 6. A higher concentration (2.5 mM) of L-carnitine prevented the degeneration of oocytes in IVG, while the survivability rate of oocytes also increased significantly in 2.5 mM of L-carnitine than the 1.875 mM group.

### 3.3. Formation of Antrum-like Structures

**Figure 2:**
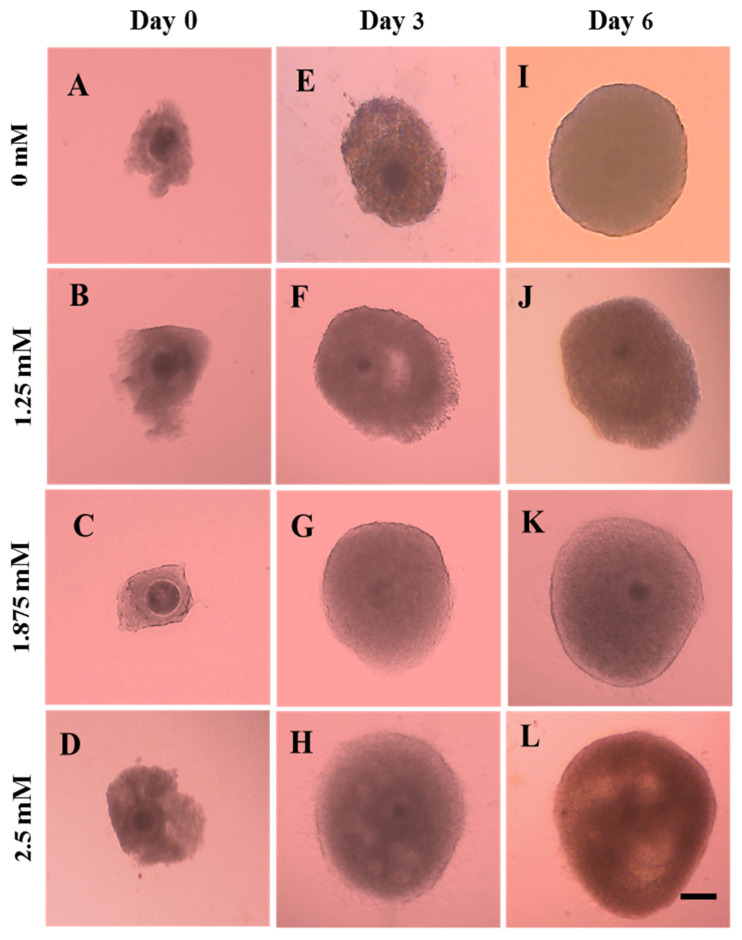
Morphology of oocyte-granulosa cell complexes cultured with 0, 1.25, 1.875 and 2.5 mM of L-carnitine on day 0 (**A**–**D**), day 3 (**E**–**H**) and day 6 (**I**–**L**). The complexes in the medium supplemented with L-Carnitine exhibited a large antrum-like structure (K and L) on Day 6. The scale bar represents 100 µm.

**Figure 3:**
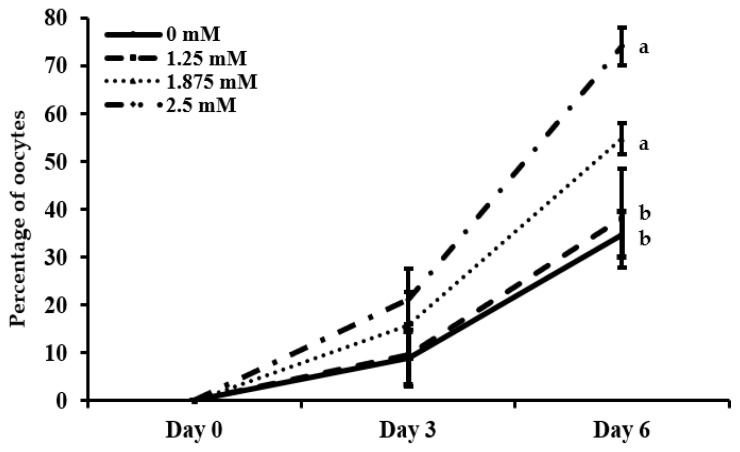
Effect of L-carnitine on antrum formation during in vitro growth culture. Formation of antrum like structures was examined on Day 0, Day 3 and Day 6 of the culture period. Different types of lines indicate different doses of L-carnitine (0, 1.25, 1.875 and 2.5 mM). Data are shown as average percentage from at least five replicated cultures. The letters “a,b” denote significantly different values (*p* < 0.05).

In the present study, the formation of extracellular space in the granulosa layer–antrum-like structures were observed on day 3 and 6 in different treatment groups of L-carnitine (Figure 2). After three days of in vitro growth culture, OGCs developed and created an extracellular space adjacent to the oocytes. They generated an antrum-like shape progressively until the end of culture. After 6 days of IVG culture, the OGCs showed 34.6%, 38.1%, 54.7% and 74.0% antrum-like structures in 0, 1.25, 1.875 and 2.5 mM of L-carnitine-supplemented groups, respectively (Figure 3). The numbers of the antrum-like structure were significantly (*p* < 0.05) higher in 2.5 mM of the L-carnitine group than the 0- and 1.25-mM groups, although the values did not differ between 1.875- and 2.5 mM-supplemented groups.

### 3.4. L-Carnitine Promotes Maturation of In Vitro Grown Oocytes

In this study, the nuclear maturation of in vitro grown oocytes was assessed and compared with that of in vivo fully grown oocytes (Table 2). Both in vivo and in vitro grown oocytes showed cumulus cell expansion after the maturation culture and some of them reached the MII stage. A significantly higher proportion of in vivo fully grown oocytes (66.6%) reached the MII stage than the in vitro grown oocytes. However, 1.25, 1.875 and 2.5 mM of L-carnitine-treated in vitro-grown oocytes showed a significantly higher rate of MII oocytes (13.6%, 25.9% and 33.3%) than the control (0%) group. Moreover, no oocytes reached the MII stage from the control (0 mM) group. Significantly higher proportions of oocytes at the DK stage were found in the control than 1.875 and 2.5 mM of L-carnitine-supplemented groups. The percentages of DK decreased gradually along with the increased concentrations of L-carnitine. Meanwhile, a lower (*p* < 0.05) percentage of degeneration was observed in 1.875 and 2.5 mM of L-carnitine-treated groups than the control group.

## 4. Discussion

In mammalian fetal ovaries, the oogonia enter into meiosis and remain arrested at Prophase I. At that time, the oocytes enlarge several folds. This process is called “oocyte growth”. The oocytes grow from 30 µm to 120 µm in bovine and porcine [24,25,26], and to 130 µm in the buffalo [12]. Growth is the result of an increase in ribosomes and mRNAs, endoplasmic reticulum, cortical granules and proteins, and an increase in the numbers of mitochondria in oocytes [27,28,29]. The mechanisms that regulate the growth of oocytes are not known well. Several factors are thought to be involved in the growth of oocytes in mammals [30,31]. In the present study, it was found that L-carnitine enhanced the growth of buffalo oocytes in vitro. It has been reported that L-carnitine supports in vitro oocyte maturation and embryo development in porcine [32]. Here, for the first time, the present study revealed that L-carnitine increased buffalo oocyte diameters. L-carnitine treatment increases the mitochondrial activity of in vitro grown porcine oocytes [32]. The mitochondrial DNA (mtDNA) copy number increases in the oocyte across oogenesis that reflects the changing ATP demands, which is needed to orchestrate cytoskeleton and cytoplasmic reorganization during ovine oocyte growth [33]. Moreover, L-carnitine treatment helps to create favorable microenvironments for protein synthesis by increasing the GSH level and reducing the ROS level [34] involved in cytoplasmic modifications, including stimulation of mRNA gene expressions and transcription factors [35]. In the present study, it is thought that the L-carnitine promotes mitochondrial activities which enhance cytoskeleton and cytoplasmic reorganization, as well as transcription abundance that ultimately supports the growth of oocytes. 

During this growth phase, the oocytes also acquire the competence to resume meiosis and reach the metaphase II (MII). Here, we found that buffalo oocytes grown with the supplementation of L-carnitine reached the MII stage, whereas oocytes grown without L-carnitine did not mature up to the MII. Lipid droplets are located with the mitochondria and the smooth endoplasmic reticulum of oocytes [36]. Fatty acids are a class of lipids that are stored intracellularly as triglycerides within the lipid droplets. Beta-oxidation of fatty acids generates ATP as a source of energy upon demand. Three-dimensional growth culture of mouse secondary follicles with the supplementation of L-carnitine did not alter the growth or differentiation of follicles [37]. However, it has been found that L-carnitine supplementation significantly increases beta-oxidation, and markedly improves oocyte maturation, fertilization and blastocyst development. The role of L-carnitine depends on cellular lipid content, mitochondrial activity and fatty acids profile in the oocyte cytoplasm [38]. Beta-oxidation is required for achieving the developmental competence of oocytes [39]. It has been reported that oocytes use lipids as an oxidative substrate for nuclear and/or cytoplasmic maturation [40,41,42]. Several studies have revealed that L-carnitine can enhance the competence of oocyte development in mammals [43]. In contrast, Marin et al. (2022) reported that L-carnitine supplementation during maturation culture of buffalo oocytes impaired oocyte development in the presence of fetal bovine serum [44]. In their study, L-carnitine increased oocyte competence in the absence of FBS. In the present study, supplementation of L-carnitine in growth culture medium increased the maturation rate of in vitro grown buffalo oocytes. Since L-carnitine increased beta-oxidation [37], and beta-oxidation ensured nuclear and/or cytoplasmic maturation of oocytes [40,41,42], it is reasonable to conclude that L-carnitine supports the nuclear and/or cytoplasmic maturation of oocytes in vitro.

Here, the formation of antrum-like structures inside the OGCs increased in L-carnitine supplemented culture media. Several factors have been recognized which regulate the formation of the follicular antrum [45]. Shen et al. reported that pig oocyte-cumulus-granulosa cell complexes form antrum in the presence of oocytes, although they did not form these structures in the absence of oocytes [46]. This indicates that certain factors produced from oocytes induce the formation of an antrum-like structure in the culture system. Later, Alam et al. reported that in the absence of oocytes, bovine granulosa cell complexes also did not form antrum-like structures, whereas supplementation of culture medium with oocyte-derived growth differentiation factor 9 (GDF9) and/or bone morphogenetic protein 15 (BMP15) induced the formation of antrum-like structures [47]. Acetyl-L-carnitine supplementation increased GDF9 and BMP15 secretion from buffalo oocytes and thereby improved embryonic development [17]. In the present study, it is thought that supplementation of culture medium with L-carnitine increased the secretion of oocyte-derived GDF9 and BMP15, which, in turn, promoted the formation of antrum-like structures by the OGCs.

L-carnitine prevented the degeneration of buffalo oocytes during in vitro growth culture. Reactive oxygen species (ROS) are generated at the time of collecting, handling or culturing oocytes in an artificial environment, which are responsible for increasing intracellular ROS levels [48,49]. It is well known that a high level of ROS causes lipid peroxidation of the cell membrane [50,51], enhances DNA fragmentation, and also influences RNA transcription and protein synthesis [34]. All of these activities are responsible for poor in vitro development and apoptosis of cells [34]. A number of studies found that L-carnitine reduced ROS in buffalo [17], mouse [18], bovine [52], porcine [53] and sheep [54] oocytes, thereby preventing oxidative damage and degeneration of oocytes. Thus, it is reasonable to conclude that L-carnitine supports the nuclear and/or cytoplasmic maturation of buffalo oocytes in vitro by increasing beta-oxidation. Similarly, reduced granulosa cell apoptosis and improved meiotic competence and mitochondrial activity of porcine growing oocytes were recorded when cultured with L-carnitine [20]. In the present study, it is reasonable to conclude that L-carnitine reduced ROS and prevent apoptosis of granulosa cells, thus preventing the degeneration of oocytes during growth culture. 

## 5. Conclusions

Supplementation of L-carnitine during in vitro growth culture enhanced the growth and maturation of buffalo oocytes. L-carnitine supplementation increased the formation of antrum-like structures and prevented the degeneration of oocytes. 

## Figures and Tables

**Table 1:** Inhibitory effect of L-carnitine on degeneration of in vitro grown buffalo oocytes.

Concentrations of L-Carnitine (mM)	Numbers of Oocytes Examined	Percentages (%) of Oocytes Degenerated		
		Day 0	Day 3	Day 6
0	40	0	45 ± 3.7 ^a^	55 ± 2.3 ^a^
1.25	40	0	40 ± 4.7 ^a^	45 ± 7.7 ^ab^
1.875	40	0	30 ± 2.2 ^a^	32.5 ± 2.2 ^b^
2.5	40	0	7.5 ± 3.2 ^b^	10 ± 2.6 ^c^

Oocyte-granulosa cell complexes from early antral follicles (1.0–1.5 mm in diameter) were collected and subjected to growth culture for 6 days with 0, 1.25, 1.875 and 2.5 mM of L-carnitine. Data are shown in mean ± SEM based on the percentages of each replicate. Percentages were calculated on the basis of total number of oocytes degenerated from the total number of oocytes examined. ^a–c^ Values with different superscripts within the same column differ significantly (*p* < 0.05).

**Table 2:** Effect of L-carnitine on nuclear maturation of in vitro grown buffalo oocytes.

Oocytes	Concentrations of L-Carnitine (mM)	Numbers of Oocytes Examined		Percentages (%) of Oocytes at Different Stage of Meiosis			
		In Vitro Growth	In Vitro Maturation	Diakinesis	Metaphase I	Metaphase II	Degenerated Oocytes
In vitro grown	0	40	18	55.5 ± 6.7 ^a^	22.2 ± 8.7	0	22.2 ± 8.3 ^a^
	1.25	40	22	45.4 ± 3.2 ^ab^	31.8 ± 8.1	13.6 ± 6.1 ^bc^	9 ± 9.6 ^ab^
	1.875	40	27	25.9 ± 4 ^bc^	44.4 ± 5.4	25.9 ± 7.1 ^b^	3.7 ± 3.3 ^b^
	2.5	40	36	19.4 ± 2.7 ^c^	44.4 ± 6.2	33.3 ± 4.6 ^b^	2.7 ± 3.3 ^b^
In vivo grown	_	_	30	10 ± 6.6 ^c^	23.3 ± 8.1	66.6 ± 7.1 ^a^	0

Oocyte-granulosa cell complexes from early antral follicles (1.0–1.5 mm in diameter) were collected and subjected to in vitro growth for 6 days. In vitro grown oocytes were transferred to IVM medium for in vitro maturation for 24 h. Data are shown in mean ± SEM based on the percentages of each replicate. Percentages were calculated on the basis of total number of oocytes in each stage of meiosis from the total number of oocytes examined. ^a–c^ Values with different superscripts within the same column differ significantly (*p* < 0.05).

## Data Availability

Data is contained within the article.

## References

[1] Warriach D.M., Mcgill R.D., Bush W.P.C., Chohan K.R. (2015). A review of recent developments in buffalo reproduction. Asian-Australas. J. Anim. Sci..

[2] Perera B.M. (2008). Reproduction in domestic buffalo. Reprod. Domest. Anim..

[3] El-Wishy A.B. (2007). The postpartum buffalo II. Acyclicity and anestrus. Anim. Reprod. Sci..

[4] Saraswat C.S., Purohit G.N. (2016). Repeat breeding: Incidence, risk factors and diagnosis in buffaloes. Asian. Pac. J. Reprod..

[5] Misra A.K. (1997). Application of biotechnologies to buffalo breeding in India. Third Course on Biotechnology of Reproduction in Buffaloes.

[6] Zicarelli L. (1997). Superovulatory response in buffaloes bred in Italy. Third Course on Biotechnology of Reproduction in Buffaloes.

[7] Yamamoto K., Otoi T., Koyama N., Horikita N., Tachikawa S., Miyano T. (1999). Development to live young from bovine small oocytes after growth, maturation and fertilization in vitro. Theriogenology.

[8] Miyano T. (2003). Bringing up small oocytes to eggs in pigs and cows. Theriogenology.

[9] Hirao Y., Itoh T., Shimizu M., Iga K., Aoyagi K., Kobayashi M., Kacchi M., Hoshi M., Takenouchi N. (2004). In vitro growth and development of bovine oocyte-granulosa cell complexes on the flat substratum: Effects of high polyvinylpyrrolidone concentration in culture medium. Biol. Reprod..

[10] Huang W., Kang S.S., Nagai K., Yanagawa Y., Takahashi Y., Nagano M. (2016). Mitochondrial activity during pre-maturational culture in in vitro-grown bovine oocytes is elated to maturational and developmental competences. Reprod. Fertil. Dev..

[11] Fair T., Hyttel P., Greve T. (1995). Bovine oocyte diameter in relation to maturational competence and transcriptional activity. Mol. Reprod. Dev..

[12] Yousaf M.R., Chohan K.R. (2003). Nuclear morphology, diameter and meiotic competence of buffalo oocytes relative to follicle size. Reprod. Fertil. Dev..

[13] Alam M.H., Lee J., Miyano T. (2018). Inhibition of PDE3A sustains meiotic arrest and gap junction of bovine growing oocytes in in vitro growth culture. Theriogenology.

[14] Ye J., Li J., Yu Y., Wei Q., Deng W., Yu L. (2010). L-carnitine attenuates oxidant injury in HK-2 cells via ROS-mitochondria pathway. Regul. Pept..

[15] Ferguson E.M., Leese H.J. (2006). A potential role for triglyceride as an energy source during bovine oocyte maturation and early embryo development. Mol. Reprod. Dev..

[16] Somfai T., Kaneda M., Akagi S., Watanabe S., Haraguchi S., Mizutani E., Dang-Nguyen T.Q., Geshi M., Kikuchi K., Nagai T. (2011). Enhancement of lipid metabolism with L-carnitine during in vitro maturation improves nuclear maturation and cleavage ability of follicular porcine oocytes. Reprod. Fertil. Dev..

[17] Xu H.Y., Yang X.G., Lu S.S., Liang X.W., Lu Y.Q., Zhang M., Lu K.H. (2018). Treatment with acetyl-l carnitine during in vitro maturation of buffalo oocytes improves oocyte quality and subsequent embryonic development. Theriogenology.

[18] Zare Z., Masteri F.R., Salehi M., Piryaei A., Ghaffari N.M., Fadaei F.F., Mohammadi M., Dehghani-Mohammadabadi M. (2015). Effect of L-carnitine supplementation on maturation and early embryo development of immature mouse oocytes selected by brilliant cresyle blue staining. J. Assist. Reprod. Genet..

[19] Gulcin I. (2006). Antioxidant and antiradical activities of L-carnitine. Life Sci..

[20] Hashimoto S., Miyata Y., Yamanaka M., Morimoto Y. (2008). L-carnitine decreased the apoptosis of granulosa cells and improved the meiotic competence of porcine growing oocytes. Reprod. Domest. Anim..

[21] Islam M.N., Alam M.H., Khatun A., Akter I., Modak A.K., Hashem M.A., Moniruzzaman M. (2020). Effects of stem cell factor on in vitro growth of buffalo oocytes. Theriogenology.

[22] Taketsuru H., Hirao Y., Takenouchi N., Iga K., Miyano T. (2011). Effect of androstenedioneon the growth and meiotic competence of bovine oocytes from early antral follicles. Zygote.

[23] Motlik J., Johnsen K.H.H., Fulka J. (1978). Breakdown of the germinal vesicle in bovine oocytes cultivated in vitro. J. Exp. Zool..

[24] Hyttel P., Fair T., Callesen H., Greve T. (1997). Oocyte growth, capacitation and final maturation in cattle. Theriogenology.

[25] Izumi T., Sakakida S., Nagai T., Miyamoto H. (2003). Allometric study on the relation between the growth of preantral and antral follicles and that of oocytes in bovine ovaries. J. Reprod. Dev..

[26] Shen X., Miyano T., Kato S., Miyamoto H., Manabe N. (1998). In vivo and in vitro antrum formation of pig ovarian follicles. Reproductive Biology Update.

[27] Gosden R.G. (2002). Oogenesis as a foundation for embryogenesis. Mol. Cell. Endocrinol..

[28] Picton H.M., Harris S.E., Muruvi W., Chamber E.L. (2008). The in vitro growth and maturation of follicles. Reproduction.

[29] Bavister B.D., Squirrell J.M. (2000). Mitochondrial distribution and function in oocytes and early embryos. Hum. Reprod..

[30] Eppig J.J. (2001). Oocyte control of ovarian follicular development and function in mammals. Reproduction.

[31] Moniruzzaman M., Miyano T. (2010). Growth of primordial oocytes in neonatal and adult mammals. J. Reprod. Dev..

[32] Hashimoto S. (2009). Application of in vitro maturation to assisted reproductive technology. J. Reprod. Dev..

[33] Cotterill M., Harris S.E., Collado F.E., Lu J., Huntriss J.D., Campbell B.K., Picton H.M. (2013). The activity and copy number of mitochondrial DNA in ovine oocytes throughout oogenesis *in vivo* oocyte maturation in vitro. Mol. Hum. Reprod..

[34] Takahashi M., Keicho K., Takahashi H., Ogawa H., Schultz R.M., Okano A. (2000). Effect of oxidative stress on development and DNA damage in in-vitro cultured bovine embryos by comet assay. Theriogenology.

[35] Lee E., Lee S.H., Kim S., Jeong Y.W., Kim J.H., Koo O.J., Park S.M., Hashem M., Hossein M., Son H.Y. (2006). Analysis of nuclear reprogramming in cloned miniature pig embryos by expression of Oct-4 and Oct-4 related genes. Biochem. Biophys. Res. Commun..

[36] Nagano M., Katagiri S., Takahashi Y. (2006). ATP content and maturational/developmental ability of bovine oocytes with various cytoplasmic morphologies. Zygote.

[37] Dunning K.R., Akison L.K., Russell D.L., Norman R.J., Robker R.L. (2011). Increased beta- oxidation and improved oocyte developmental competence in response to l-carnitine during ovarian in vitro follicle development in mice. Biol. Reprod..

[38] Baldoceda L., Gagné D., Ferreira C.R., Robert C. (2016). Genetic influence on the reduction in bovine embryo lipid content by l-carnitine. Reprod. Fertil. Dev.

[39] Dunning K.R., Cashman K., Russell D.L., Thompson J.G., Norman R.J., Robker R.L. (2010). Beta-oxidation is essential for mouse oocyte developmental competence and early embryo development. Biol. Reprod..

[40] Cetica P., Pintos L., Dalvit G., Beconi M. (2002). Activity of key enzymes involved in glucose and triglyceride catabolism during bovine oocyte maturation in vitro. Reproduction.

[41] Kim J.Y., Kinoshita M., Ohnishi M., Fukui Y. (2001). Lipid and fatty acid analysis of fresh and frozen-thawed immature and in vitro matured bovine oocytes. Reproduction.

[42] Rieger D., Loskutoff N. (1994). Changes in the metabolismof glucose, pyruvate, glutamine and glycine during maturation of cattle oocytes. J. Reprod. Fertil..

[43] Li J., Liu L., Weng J., Yin T.L., Yang J., Feng H.L. (2021). Biological roles of l-carnitine in oocyte and early embryo development. Mol. Reprod. Dev..

[44] Marin D.F.D., da Costa N.N., Santana P.D.P.B., de Souza E.B., Rolim Filho S.T., da Silva Cordeiro M., Ohashi O.M. (2020). Influence of L-carnitine on lipid metabolism of buffalo cumulus-oocyte complexes matured in either fetal bovine serum or fatty acid-free bovine serum albumin. Theriogenology.

[45] Rodgers R.J., Irving-Rodgers H.F. (2010). Formation of the ovarian follicular antrum and follicular fluid. Biol. Reprod..

[46] Shen X., Miyano T., Kato S. (1998). Promotion of follicular antrum formation by pig oocytes in vitro. Zygote.

[47] Alam M.H., Lee J., Miyano T. (2018). GDF9 and BMP15 induce development of antrum-like structures by bovine granulosa cells without oocytes. J. Reprod. Dev..

[48] Fujitani Y., Kasai K., Ohtani S., Nishimura K., Yamada M., Utsumi K. (1997). Effect of oxygen concentration and free radicals on in vitro development of in vitro-produced bovine embryos. J. Anim. Sci..

[49] Koo O.J., Jang G., Kwon D.K., Kang J.T., Kwon O.S., Park H.J., Kang S.K., Lee B.C. (2008). Electrical activation induces reactive oxygen species in porcine embryos. Theriogenology.

[50] Nasr-Esfahani M.H., Aitken J.R., Johnson M.H. (1990). Hydrogen peroxide levels in mouse oocytes and early cleavage stage embryos developed in vitro or in vivo. Development.

[51] Noda Y., Matsumoto H., Umaoka Y., Tatsumi K., Kishi J., Mori T. (1991). Involvement of superoxide radicals in the mouse two-cell block. Mol. Reprod. Dev..

[52] Sovernigo T.C., Adona P.R., Monzani P.S., Guemra S., Barros F., Lopes F.G., Leal C. (2017). Effects of supplementation of medium with different antioxidants during in vitro maturation of bovine oocytes on subsequent embryo production. Reprod. Domest. Anim..

[53] You J., Lee J., Hyun S.H., Lee E. (2012). L-carnitine treatment during oocyte maturation improves in vitro development of cloned pig embryos by influencing intracellular glutathione synthesis and embryonic gene expression. Theriogenology.

[54] Mishra A., Reddy I.J., Gupta P.S., Mondal S. (2016). L-carnitine mediated reduction in oxidative stress and alteration in transcript level of antioxidant enzymes in sheep embryos produced in vitro. Reprod. Domest. Anim..

